# Studies on the Processes of Electron Capture and Clustering of Benzyl Chloride by Ion Mobility Spectrometry

**DOI:** 10.3390/molecules26154562

**Published:** 2021-07-28

**Authors:** Izabela Wolańska, Edyta Budzyńska, Jarosław Puton

**Affiliations:** Faculty of Advanced Technologies and Chemistry, Military University of Technology, ul. Gen. Sylwestra Kaliskiego 2, 00-908 Warsaw 46, Poland; izabela.wolanska@wat.edu.pl (I.W.); edyta.budzynska@wat.edu.pl (E.B.)

**Keywords:** ion–molecule reactions, dissociative electron capture, cluster ions, reaction chemistry, ion mobility spectrometry

## Abstract

This paper presents the results of the study on the course of the benzyl chloride (BzCl) ionization process in a drift tube ion mobility spectrometer (DT IMS) in which nitrogen was used as the carrier gas. BzCl ionization follows the dissociative electron capture mechanism. The chloride ions produced in this process take part in the formation of cluster ions. Using DT IMS allows for estimation of the value of the electron attachment rate for BzCl and the equilibrium constant for the cluster ion formation. The basic experimental method used in this work was to analyze drift time spectra obtained for the introduction of the sample to the spectrometer with the drift gas. The theoretical interpretation of the results is based on the mathematical description of the ion transport. This description takes into account the phenomenon of diffusion, as well as the processes of formation and dissociation of ionic clusters occurring during the movement of ions in the drift section.

## 1. Introduction

Ion mobility spectrometry (IMS) is an analytical technique based on the study of ion movement in the gas phase under the influence of an electric field [1]. The important application of IMS is chemical analysis and, in particular, the *on-site* detection of hazardous substances. Currently, the features of IMS that are the basis of explosives [2,3] and warfare agents’ detection [4] are the apparatus’s simplicity, speed, and relative reliability of detection.

In addition to analytical applications, IMS is often used in fundamental research. Their main subjects are phenomena related to the formation of ions from various substances, as well as the charge transport in gases. Standard drift tube ion mobility spectrometers (DT IMS) can be used in studies that allow estimation of the rate constants of ion–molecular reactions [5], as well as the thermodynamic parameters of these processes [6,7,8]. In studies of the kinetics and thermodynamics of ionization, the processes taking place in the drift region are most often considered. This is due to the fact that in this region, there is a homogeneous electric field in which the time of interaction between the ions and neutral molecules is strictly defined. In the drift region, it is possible to explore the decomposition processes of ions generated in the reaction region of the detector [6]. Many more possibilities are ensured by introducing the sample into the drift region along with the drift gas. The drift time spectra obtained in such a way contains peaks that correspond to specific types of ions and continuous sections in which the ion current is generated by ions formed or dissociated inside the drift region.

The analysis of the time dependence of the ionic current for these sections allows for the determination of kinetic constants [5,9,10]. When the lifetime of product ions formed as a result of reactions taking place in the drift region is much shorter than the characteristic drift time, the peak position depends on the concentration of the sample introduced into the drift gas [11]. Based on the measurement of changes in peak position, it is possible to estimate the equilibrium constant for the cluster ions formation [12].

A specific type of ionization of some compounds is the attachment of electrons with low (thermal) energy. This process is used in electron capture detectors (ECDs) applied in gas chromatography [13]. Electron attachment also occurs in ion mobility spectrometers when nitrogen is used as a carrier gas. The most commonly observed process is the dissociative electron attachment (DEA), in which the attachment of an electron to a molecule is associated with its dissociation [14]. A typical example of such a process is the interaction of a thermal electron with a chloro-organic compound, which produces a chloride ion:

(1) $$e^{-}+\mathrm{BCl}\overset{\;k_{EC}\;}{\to }B+{\mathrm{Cl}}^{-}$$

where *k_EC_* is the electron attachment rate constant. The values of the electron attachment rate constant vary for different compounds and depend on the electrons’ energy. The use of DT IMS to determine the electron attachment rate for chloroform and tetrachloromethane molecules was proposed by Tabrizchi [15]. The same method was used in tests for chlorobenzenes [16], chlorobromomethane [17], and oxygen [18]. Chloride ions very often form cluster ions with molecules with a positive electron affinity. Usually, the stability of the clusters is not very high, and, especially at higher temperatures, they dissociate. The process of clusters formation and dissociation can be described by Equation (2):

(2) $$M+{\mathrm{Cl}}^{-}\overset{\;k_{c}/k_{d}\;}{↔}M\cdot {\mathrm{Cl}}^{-}$$

where *k_c_* is the reaction rate constant for generation of M∙Cl^−^ clusters, and *k_d_* is the constant of their dissociation. Substances that generate chloride ions as a result of dissociative electron attachment or dissociative charge transfer are used as dopants for the detection of explosives [2,19]. The analysis of ions clustering in the case of isoflurane and enflurane can be found in [20]. Experimental data and calculations of thermodynamic parameters presented in this paper show that it is possible to create more complex structures consisting of a chloride ion and two analyte molecules.

The aim of our work was to conduct research that will characterize both the electron capture process and the cluster ions formation. It can be noticed in [16] that sometimes these two processes simultaneously influence the obtained measurement data (drift time spectra). The theoretical model of charge transport in the drift region included in the paper allows for the prediction of the shape of drift time spectra and assessment of the influence of ion–molecule reactions on the accuracy of the determination of the electron attachment rate. Benzyl chloride (BzCl) was selected as the test substance. Preliminary studies have shown that reactions (1) and (2) are observed during the chemical ionization of this compound in nitrogen.

## 2. Materials and Methods

The DT IMS used in this work was constructed at the Institute of Chemistry in Military University of Technology (Warsaw, Poland). The detector is made of a set of stainless-steel rings separated by glass insulators. The reaction and drift regions’ lengths are 5.7 and 6.1 cm, respectively. The diameters of the reaction and drift regions are 2.2 cm and 3.6 cm. The gas ionization takes place under the influence of beta radiation emitted from a radioactive source ^63^Ni (15 MBq). If the carrier gas is nitrogen, the primary ionization leads to the formation of positive ions (N_2_^+^) and electrons, which may be involved in the process of dissociative attachment to the sample molecules. Ions and electrons generated in the reaction region are injected into the drift section by a Bradbury–Nielsen shutter grid. All measurements were performed for a grid opening time of 0.15 ms and electric field intensity of 251 V·cm^−1^ in the drift region. Drift spectra were recorded with a digital oscilloscope using a signal averaging of 128 samples. The flows of carrier and drift gas were the same and equal to 0.5 L·min^−1^, and purified nitrogen (99.999%, MULTAX) was used for both cases. The DT IMS temperature ranged from 318 to 363 K. The tests were performed for benzyl chloride (BzCl) (99%, CAS: 100-44-7, Sigma-Aldrich, Burlington, MA, USA).

**Figure 1:**
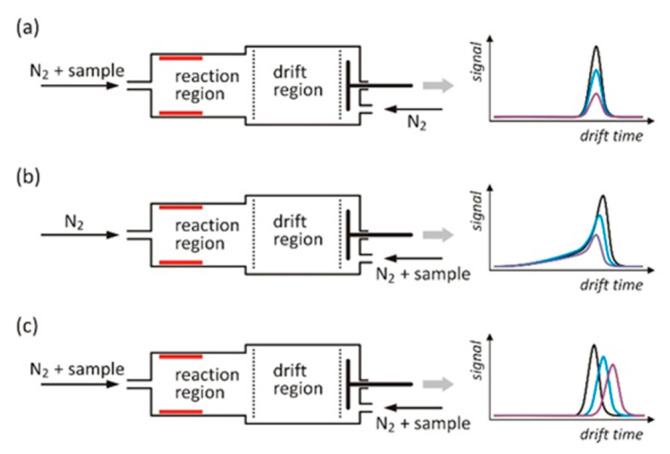
Different variants of the sample introduction method into the IMS detector: (**a**) Measurements of drift times and ion mobilities; (**b**) measurement of the electron attachment rate; (**c**) peak shift studies from which the equilibrium constant for the clustering reaction was determined. The drift time spectra shown on the right correspond to the different concentrations of the sample introduced into the carrier or drift gas.

The concentration of BzCl was precisely controlled. The gas mixtures generator system was similar to that presented in [21]. The source of BzCl vapors were permeation standards with emissions from 5.2 × 10^−6^ to 1.0 × 10^−5^ g·min^−1^. Dilution of the sample stream was controlled by a pneumatic system equipped with six mass flow controllers (Brooks SLA5850 and GFA40) with two control modules (Brooks 0254) (Brooks Instrument, Hatfield, PA, USA). Three types of experiments were performed that differed in the way of the sample introduction into the detector. Measurements of the ions’ drift time spectra, which were used to determine the drift times and mobility used in further calculations, were made with the introduction of the sample from the carrier gas inlet (Figure 1a).

The determination of the electron attachment rate constants was carried out when the sample was introduced with the drift gas, while the carrier gas was pure nitrogen (Figure 1b). The process of formation and decomposition of cluster ions was investigated in the system shown in Figure 1c. In these measurements, the sample was introduced with both carrier gas and drift gas. The concentration of the sample in the carrier gas was constant and high enough that all electrons in the reaction region were absorbed by the dissociative attachment. The concentration of the sample in the gas introduced into the drift section was variable.

Numerical calculations, the results of which are shown in the next section, were performed with the MATLAB R2020b program (MathWorks Inc., Natick, MA, USA).

### 2.1. The Theory of Charge Transport in the Drift Section of IMS Detectors

#### 2.1.1. A Simple Model Used to Determine the Electron Attachment Rate

Electron attachment rate constants were determined using the method based on the measurement of the ionic current generated in DT IMS when a sample of the test substance is introduced with the drift gas [15]. The idea behind the measurement is based on the assumption that the portion of electrons injected into the drift region generates in a very short time, producing ions that are formed in the electron capture process. These ions are then moved along the drift region toward the collector electrode, where an ion current is generated. For the substance studied in this work (BzCl), the product of dissociative electron attachment is the chloride ion (Cl^−^). The initial spatial distribution of its concentration can be easily determined from consideration of the kinetics of the electron capture reaction. The dependence of the concentration of chloride ions *n_Cl_^−^* on the distance *x* between the shutter grid and a given point in the drift section at time *t* = 0 is described by Equation (3):

(3) $$n_{{Cl}^{-}}\left(x,0\right)=k_{EC}n_{0e}n_{M}t_{g}\exp\left(-\frac{k_{EC}n_{M}x}{v_{e}}\right)+n_{{Cl}^{-},RS}\left(x,0\right)$$

where *k_EC_* is the electron attachment rate constant, *n*_0*e*_ and *n_M_* are the concentrations of electrons at the beginning of the drift region and of the analyte in the drift gas, *t_g_* is the shutter grid opening time, and *v_e_* is the electron drift velocity. The additional *n_Cl_^−^,_RS_* component present in Equation (3) is related to the fact that a certain amount of chloride ions is already generated in the reaction region and injected into the drift section during the dosing pulse. Chloride ions with mobility *K_Cl_^−^*, for which the initial concentration distribution is given by Equation (3), move along the drift section in the electric field *E* with the velocity of *v_Cl_^−^* = *K_Cl_^−^E*. Ion current *I_Cl_*^−^(*t*) measured in the collector electrode at time *t* from the end of the dosing pulse can be expressed by Equation (4):

(4) $$I_{{Cl}^{-}}\left(t\right)=A_{ce}v_{{Cl}^{-}}en_{{Cl}^{-}}\left(l_{d},t\right)=I_{cont}+I_{peak}$$

where *A_ce_* is the area of the collector electrode, *e* is the elementary charge, and *l_d_* is the length of the drift path. The concentration of chloride ions at the end of the drift section *n_Cl_^−^ (l_d_,t)* can be easily determined assuming that the shape of the concentration distribution is described by Equation (3) and taking into account the shift of the ion cloud toward the collector electrode:

(5) $$n_{{Cl}^{-}}\left(l_{d},t\right)=n_{{Cl}^{-}}\left(l_{d}-v_{{Cl}^{-}}t,0\right)$$

The components *I_cont_* and *I_peak_* in Equation (4) correspond, respectively, to chloride ions produced by electrons in the drift section and the ions injected from the reaction section. The slope of the log *I_cont_* against time is proportional to the electron attachment rate:

(6) $$ln\left(I_{cont}\left(t\right)\right)=const+\frac{k_{EC}n_{M}v_{{Cl}^{-}}t}{v_{e}}$$

#### 2.1.2. Description of the Ions’ Movement in the Drift Section, Including Diffusion and Ion–Molecule Reactions

The analysis of the experimental data obtained in the tests when the sample is introduced together with the drift gas is complex. In the drift time spectra, continuous regions as well as peaks, characterized by a variable position and width, are observed. Modeling of the drift process allows for observation of the influence of such phenomena as diffusion or ion–molecular reactions on spectra.

The shape of the concentration distribution for ions moving in the drift section changes with time. If the electric field is homogeneous, then the main causes of these changes may be diffusion broadening, as well as ion–molecule reactions. The course of these reactions depends on the composition of the drift gas and on the type of ions moving in the gas. For the reaction of formation and decomposition of cluster ions following Equation (2), the ion balance in the drift section can be described by the system of the two differential equations:

(7) $$\begin{array}{l} \frac{\partial n_{{Cl}^{-}}}{\partial t}=-k_{c}n_{M}n_{{Cl}^{-}}+k_{d}n_{M{Cl}^{-}}-K_{{Cl}^{-}}E\frac{\partial n_{{Cl}^{-}}}{\partial x}+D_{{Cl}^{-}}\frac{\partial^{2}n_{{Cl}^{-}}}{\partial x^{2}}\\ \frac{\partial n_{{MCl}^{-}}}{\partial t}=k_{c}n_{M}n_{{Cl}^{-}}-k_{d}n_{M{Cl}^{-}}+K_{M{Cl}^{-}}E\frac{\partial n_{{MCl}^{-}}}{\partial x}+D_{M{Cl}^{-}}\frac{\partial^{2}n_{{MCl}^{-}}}{\partial x^{2}} \end{array}$$

where *n_MCl_^−^* and *n_M_* are concentrations of cluster ions and sample molecules, *k_c_* and *k_d_* are the rate constants related to the Equation (2), *K_Cl_^−^* and *K_MCl_^−^* are the mobilities, and *D_Cl_^−^* and *D_MCl_^−^* are the diffusion coefficients. The initial conditions necessary for solving this system of equations are defined by the initial ion concentration distributions. It was assumed that the time *t* = 0 corresponds to the closing of the shutter grid. The initial distribution of chloride ions concentration is given by Equation (3). The distribution of the cluster ions concentration contains only the *n_MCl_^−^_,RS_* component as a result of their injection from the reaction section during the dosing pulse. The shape of the distribution of ions injected into the drift section can be approximated by the difference of error functions:

(8) $$\begin{array}{l} n_{{Cl}^{-},RS}\left(x,0\right)=0.5n_{0{Cl}^{-}}\left(erf\left(Bx\right)-erf\left(B\left(x-K_{{Cl}^{-}}Et_{g}\right)\right)\right)\\ n_{M{Cl}^{-},RS}\left(x,0\right)=0.5n_{0M{Cl}^{-}}\left(erf\left(Bx\right)-erf\left(B\left(x-K_{M{Cl}^{-}}Et_{g}\right)\right)\right) \end{array}$$

where *B* is the constant determining ion swarm broadening and *n*_0*Cl*_*^−^* and *n*_0*MCl*_*^−^* are the concentrations of the respective types of ions introduced through the opened shutter grid.

**Figure 2:**
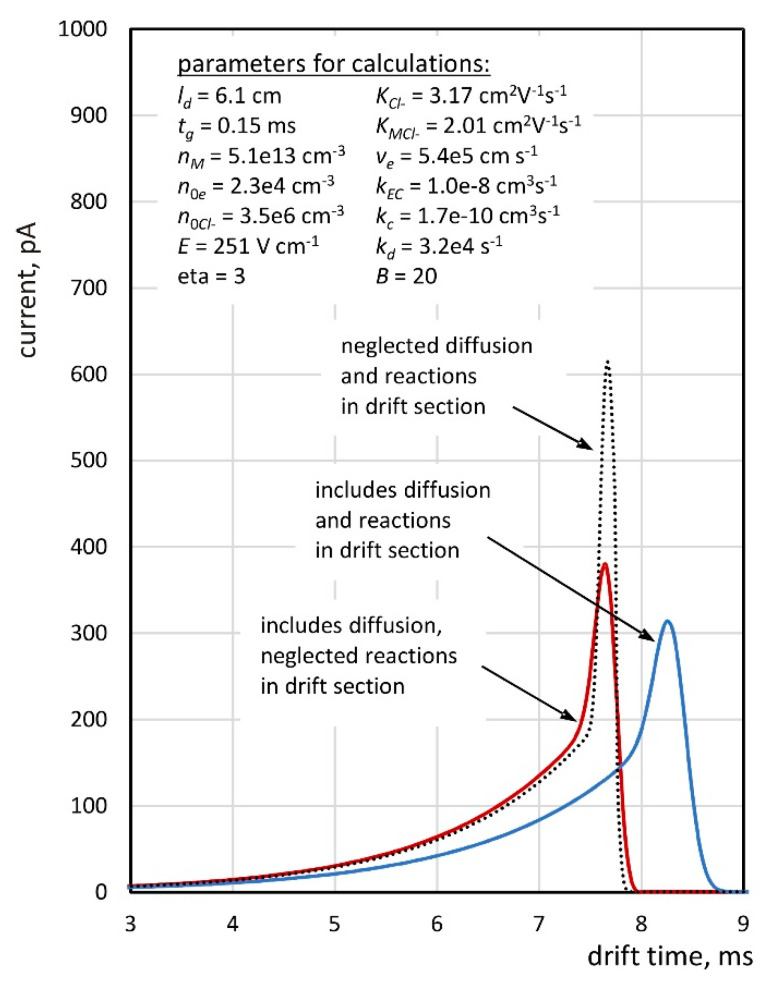
Time dependence of ionic current calculated without diffusion and ion–molecule reactions and taking into account these phenomena.

The system of Equation (7) with the initial conditions given by Equations (3) and (8) can be classified as the advection/diffusion problem. A solution to this problem for a one-dimensional case can be obtained by using the *pdepe function* (parabolic and elliptic partial differential equations) of the standard MATLAB function set. The knowledge of the time-dependent concentration distributions for both types of ions allows for the calculation of the current in the collector electrode. Examples of the dependence of this current on time are shown in Figure 2. The figure also contains values of the parameters for which the calculations were performed. These data correspond to the design parameters of the IMS detector and the mobilities measured for BzCl. The electron attachment rate constant *k_EC_* was determined experimentally (see Section 3.2), and the rate constants for the formation and decay of the cluster ions (*k_c_* and *k_d_*) were estimated in such a way that the peak shift with increasing analyte concentration corresponded to the measurement results (see Section 3.3). The MATLAB functions used for calculations are included in the Supplementary Materials. The values of the diffusion coefficients were estimated on the basis of the Nernst–Einstein relationship, taking into account the energy factor *η* [22]:

(9) $$D=\eta K\frac{k_{B}T}{e}$$

where *k_B_* is the Boltzmann constant and *T* is the temperature.

Analyzing the dependencies presented in Figure 2, it can be concluded that both diffusion and ion–molecule reactions have a significant impact on the drift time spectra. It is obvious that diffusion influences the broadening of the peak in the spectrum; however, the value of the ion current for drift times shorter than 7 ms does not differ significantly for both spectra calculated without the ion–molecule reactions. Taking these reactions into consideration results in much more noticeable effects. The peak in the spectrum is shifted toward longer drift times by a value of approximately 0.6 ms, and, moreover, its broadening is much more visible. The value of the current in the continuous section of the spectrum also goes down. Therefore, an important problem is the assessment of the accuracy of the electron attachment rate constant calculation based on Equation (6) in the case when the charge transport in the drift section takes place with an ongoing reaction. The performed calculations that are based on Equation (6) showed that the *k_EC_* values determined for all the curves in Figure 2 do not differ from each other by more than 0.7%. It requires taking into account the fact that the drift velocity of the *v_Cl_^−^* ions in Equation (6) is calculated using the drift time measured for the drift gas containing a specific concentration of the sample. It can be assumed that the above-described method for determining the electron attachment rate is accurate and insensitive to changes in the shape of the spectrum caused by ion–molecule reactions.

#### 2.1.3. Shift of the Peaks Caused by Ion–Molecule Reactions

The influence of the ion–molecule reactions on the position of the peak in the drift time spectrum can be analyzed on the basis of a simple model of the formation and decomposition of cluster ions. The shift of the peak shown in Figure 2 is related to the fact that the charge transfer along the drift section is accompanied by multiple changes to the charge carrier. In the case taken under consideration, these carriers are chloride ions Cl^-^ and M∙Cl^−^ cluster ions. The average lifetimes of these carriers, *τ_Cl_^−^* and *τ_MCl_^−^*, are related to the kinetic constants *k_c_* and *k_d_* appearing in Equation (7). The chloride ion lifetime is also a function of the sample concentration:

(10) $$\tau_{{Cl}^{-}}=\frac{1}{k_{c}n_{M}},\;\tau_{M{Cl}^{-}}=\frac{1}{k_{d}}$$

In the time equal to *τ_Cl_^−^* + *τ_MCl_^−^*, the charge is transferred to the distance *K_Cl_^−^Eτ_Cl_^−^* + *τ_MCl_^−^K_MCl_^−^E*. Based on this, the effective mobility coefficient can be calculated:

(11) $$K_{eff}=\frac{\tau_{{MCl}^{-}}K_{{MCl}^{-}}+\tau_{{Cl}^{-}}K_{{Cl}^{-}}}{\tau_{{MCl}^{-}}+\tau_{{Cl}^{-}}}=\frac{\kappa n_{M}K_{{MCl}^{-}}+K_{{Cl}^{-}}}{\kappa n_{M}+1}$$

**Figure 3:**
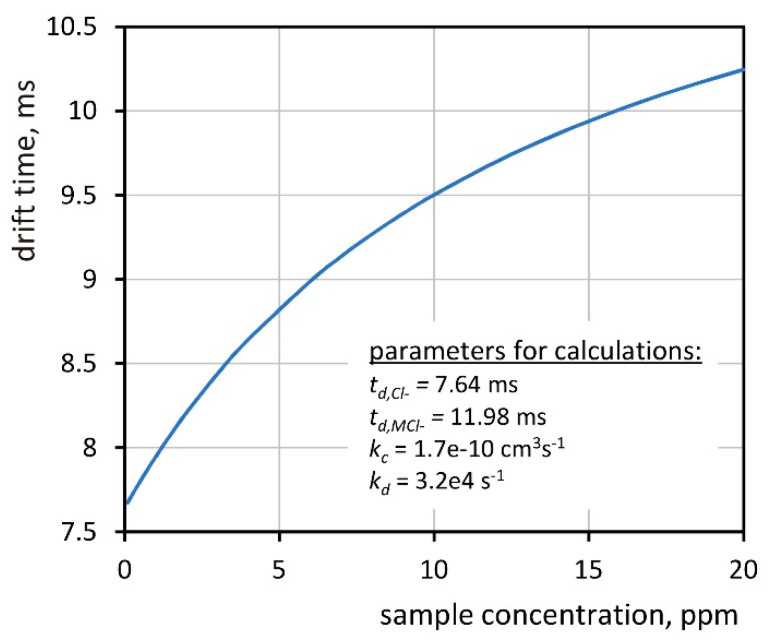
The dependence of the effective drift time on the sample concentration.

It is also possible to determine the effective drift time:

(12) $$t_{d,eff}=\frac{\left(\tau_{M{Cl}^{-}}+\tau_{{Cl}^{-}}\right)t_{{d,Cl}^{-}}t_{{d,MCl}^{-}}}{\tau_{{MCl}^{-}}t_{{d,Cl}^{-}}+\tau_{{Cl}^{-}}t_{{d,MCl}^{-}}}=\frac{\left(\kappa n_{M}+1\right)t_{{d,Cl}^{-}}t_{{d,Cl}^{-}}}{\kappa n_{M}t_{{d,Cl}^{-}}+t_{{d,MCl}^{-}}}$$

where *t_d,Cl_^−^* is the chloride ion drift time when there is no sample in the drift section, *t_d,MCl_^−^* is the cluster ion drift time, and *κ* = *k_c_/k_d_* is the ratio of the kinetic constants. An exemplary graph of the dependence of the effective drift time on the sample concentration is shown in Figure 3.

Measurement of the peak shift in the drift time spectrum allows for the estimation of the equilibrium constant *K_eq_*_(2)_ for reaction described by Equation (2):

(13) $$K_{eq\left(2\right)}=\kappa n_{0}=\frac{n_{0}}{n_{M}}\frac{t_{d,eff}/t_{{d,Cl}^{-}}-1}{1-t_{d,eff}/t_{{d,MCl}^{-}}}$$

where *n*_0_ is the number of drift gas molecules per unit volume.

## 3. Results

### 3.1. Drift Times and Reduced Mobilities

**Figure 4:**
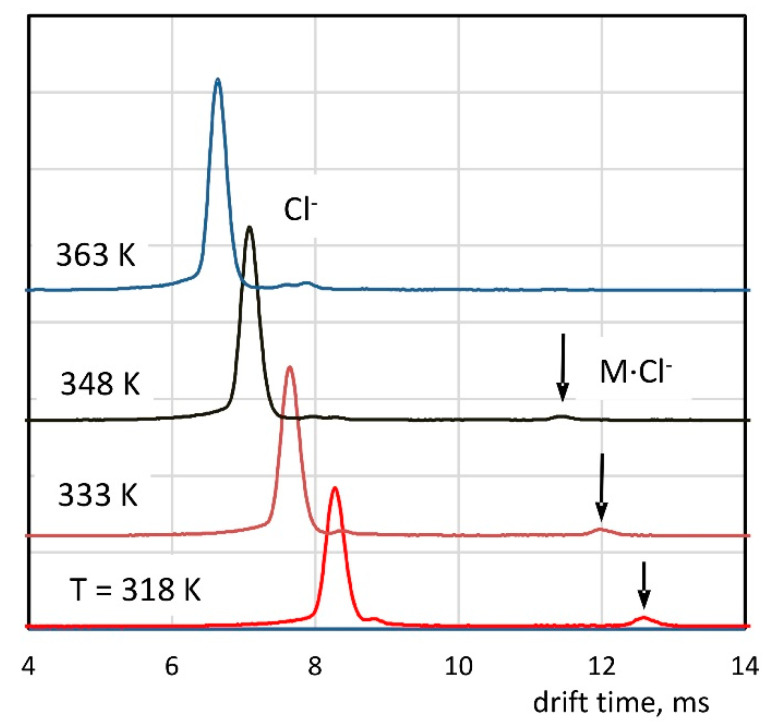
Drift time spectra with the introduction of the sample with the carrier gas.

The determination of the electron attachment rate constants and the equilibrium constant of the clustering reaction requires the knowledge of the drift times and ion velocity. The drift time spectra were analyzed when the sample was introduced into the detector along with the carrier gas (see Figure 1a). The results of these measurements are shown in Figure 4. The obtained spectra are very simple, and the dominant peaks appearing in these spectra correspond to chloride ions generated as a result of dissociative electron attachment. Spectra measured at lower temperatures also contain small peaks of cluster ions. For a temperature of 363 K, the peak of the cluster ions is not appearing. The drift times and reduced mobilities determined on the basis of the spectra are summarized in Table 1. The reduced mobility of chloride ions increases when the temperature rises. The latter is a consequence of chloride ions, which can be hydrated at temperatures at which the research was conducted, and the degree of hydration depends on the temperature [23]. The reduced mobility of the cluster ions is practically constant, suggesting that the ions are not hydrated or do not change the degree of hydration within the measurement temperature range.

### 3.2. Electron Attachment Rates

**Figure 5:**
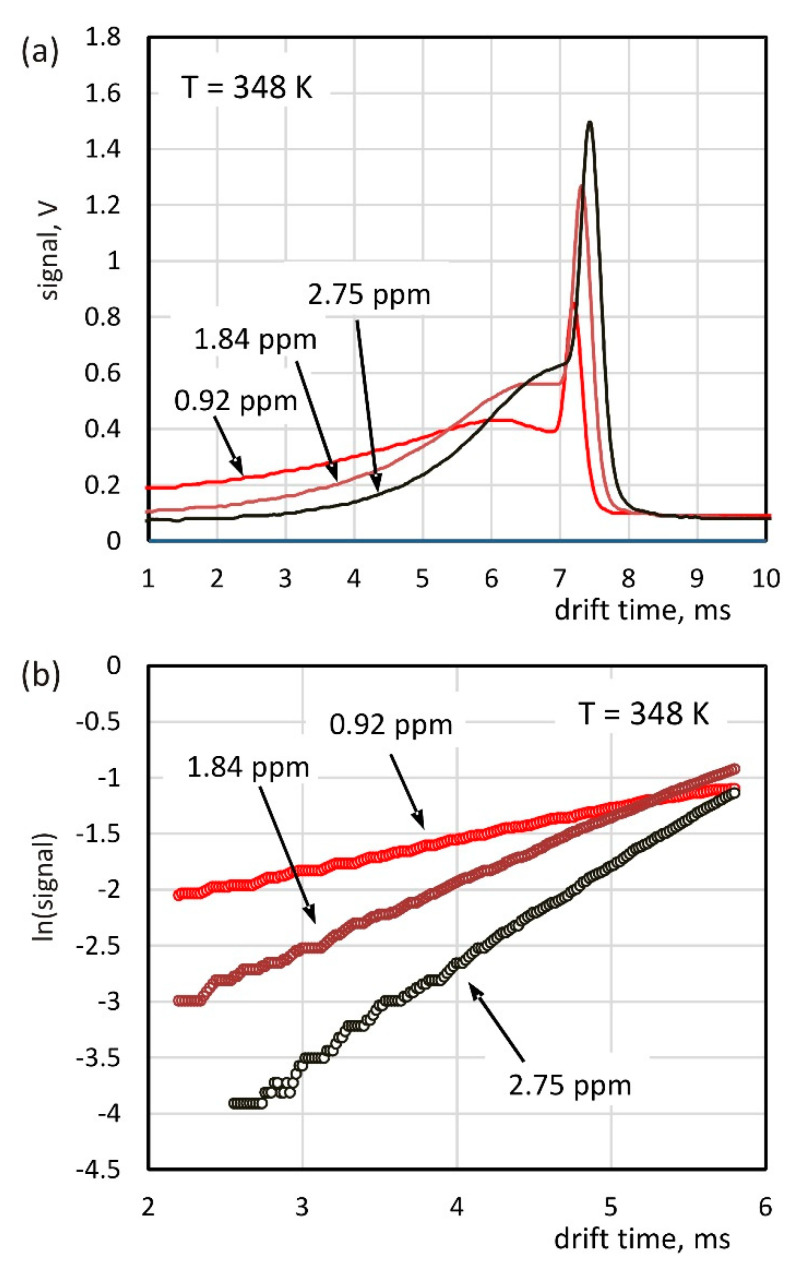
The drift time spectra measured for the introduction of the sample with drift gas (**a**) and the graph of the signal logarithm versus drift time used to determine the electron attachment rate constants (**b**).

The study of the electron capture kinetics was carried out when the sample was introduced into the drift region (Figure 1b). Examples of drift time spectra measured at 348 K are shown in Figure 5a. The shape of these spectra is similar to the theoretically determined dependencies (Figure 2); however, for the times preceding the peak (from 6 to 7 ms), a certain decrease in the ion current is observed. This effect is also noticeable in the test results shown in other works [24] and may be related to the introduction of a certain amount of pure gas into the drift section from the side of the reaction region. The graph of the dependence of the signal logarithms on time in the continuous area of the spectrum is shown in Figure 5b. The obtained dependencies can be approximated with linear functions, and their slope allows for the determination of the electron attachment rates based on Equation (6). The electron drift velocity *v_e_* was calculated on the basis of data presented in the works of Nakamura [25] and Jarvis [26]. The value of the drift velocity of the chloride ions *v_Cl_^−^* was determined on the basis of the drift time measured at a given concentration of the sample in the drift gas. It is clearly noticeable that the peaks in the spectra presented in Figure 5a are shifting toward longer drift times with increasing sample concentration. This is due to the mechanisms of the carrier change during the charge transport in the drift section discussed in the previous paragraph. All the determined values of the electron attachment rates are summarized in Table 2.

The values of the electron attachment rate constants presented in Table 2 are relatively high. The available literature does not provide data on benzyl chloride; in particular, there are no available measurement results obtained using IMS detectors. In very old works, the values of 4 × 10^−9^ cm^3^s^−1^ [27] and 5.5 × 10^−10^ cm^3^s^−1^ [28] are given, which are significantly lower than the values in Table 2. However, the conditions under which those measurements were carried out were completely different from those in the DT IMS detector. The gas mixtures used in the mentioned studies contained sulfur hexafluoride [27] and *n*-hexane [27], and the experiments were carried out at a zero value of electric field intensity. The results obtained with the IMS detector for double and triple chlorinated benzenes by Feng et al. [16] contained *k_EC_* values ranging from 7 × 10^−10^ cm^3^s^−1^ (1,4-dichlorobenzene) to 8 × 10^−9^ cm^3^s^−1^ (α,α,α-trichlorotoluene).

The obtained values of the electron attachment rates increase with temperature. Such a phenomenon is experimentally observed for many halogenated organic compounds [29]. The theoretical explanation of the phenomenon may be based on the dependence of the cross-section for dissociative capture on the energy of electrons. The maximum cross-section for benzyl chloride is at about 0.8 eV [30,31]. This energy value is higher than the energy of electrons in nitrogen under the electric field in the IMS detector. The value of the electric field in the drift section in the detector used in the tests is 251 V cm^−1^. For that field value, at the temperature of 333 K, the energy of electrons in nitrogen is equal to approximately 0.40 eV [32]. Thus, an increase in temperature causes an increase in the cross-section for the capture process.

### 3.3. Equilibrium Constants for Ion Clustering Processes

**Figure 6:**
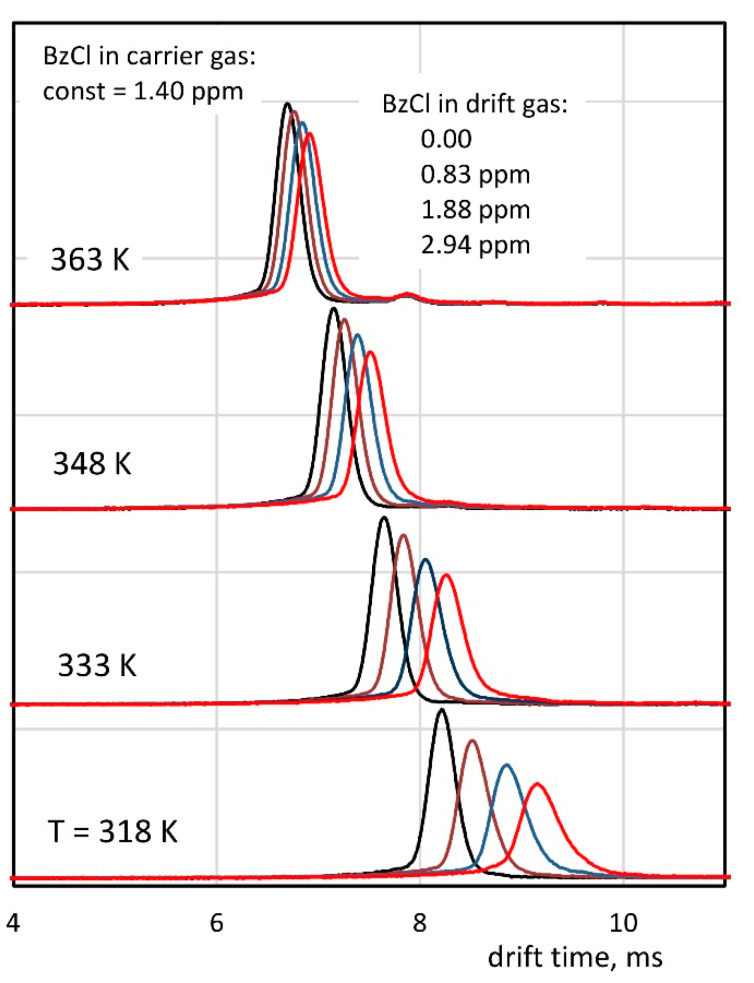
The effect of the peak shift with the introduction of the sample into the drift gas. All measurements were made with a BzCl concentration in the carrier gas of 1.40 ppm.

The drift time spectra measured with the introduction of the sample with the carrier and drift gases (see Figure 1c) are shown in Figure 6. As the BzCl concentration in the drift gas increases, an apparent shift of the peak toward greater drift times can be observed. This effect is especially noticeable at lower temperatures.

**Figure 7:**
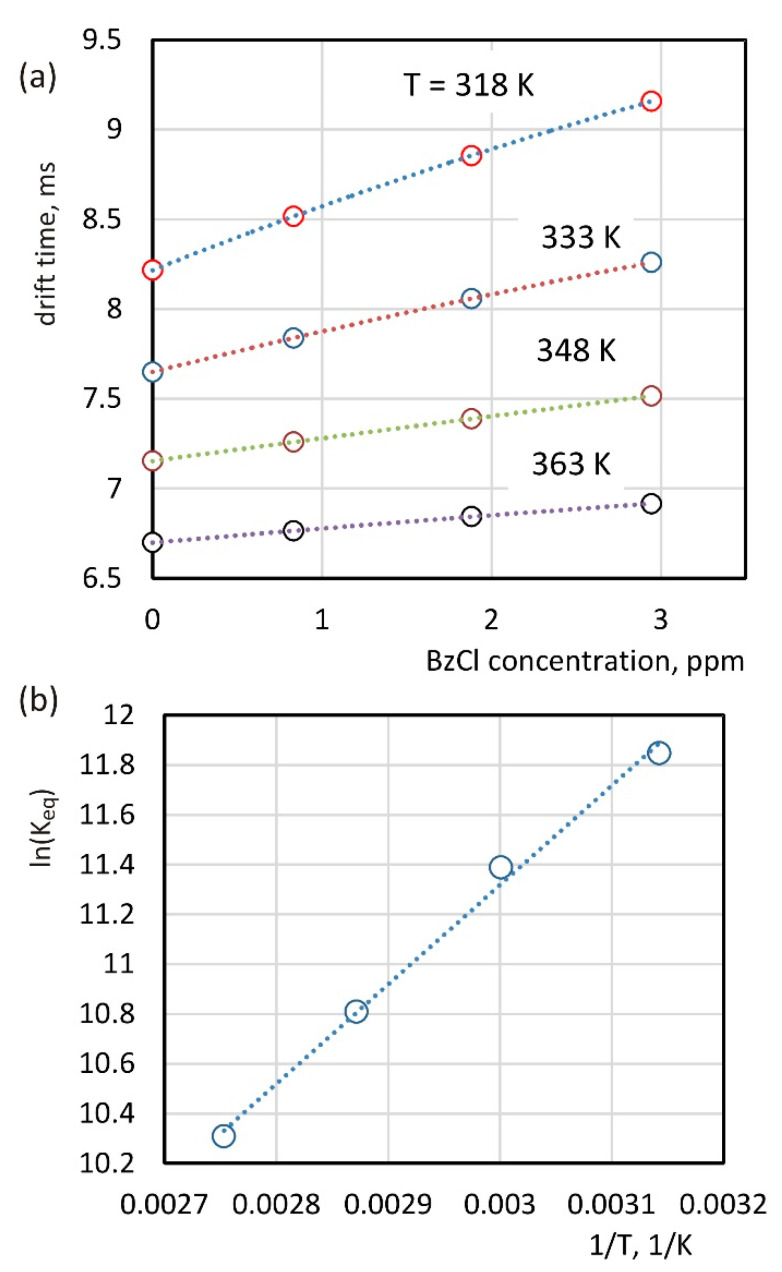
Peaks positions measured for the introduction of BzCl to the drift gas (**a**) and a plot of the logarithm of the equilibrium constant versus the reciprocal of temperature (**b**).

The position of the peak in the spectra shown in Figure 6 corresponds to the effective drift time. Its dependence on the BzCl concentration in the drift gas is shown in Figure 7a. The knowledge of the effective drift time, as well as the drift times for chloride ions and cluster ions, allows for the determination of the equilibrium constants using Equation (13). The value of the cluster drift time *t_d,MCl_^−^* for the temperature of 363 K was calculated for reduced mobility equal to 1.65 cm^2^V^−1^s^−1^. All the other values of drift times values needed to perform the calculations were taken from Table 1. The calculated values of the equilibrium constants are summarized in Table 3. The dispersion of these values is small. The relative standard deviation at a constant temperature for different BzCl concentrations does not exceed 2.0%. The dependence between the drift times and the equilibrium constant was also analyzed by Izadi et al. [12]. They determined the equilibrium constant for the process of formation/decomposition of dimer ions based on the slope of the effective drift time dependence on the sample concentration. The calculations for the results presented in this paper were based on other mathematical methods and were performed for different values of analyte concentrations at four different temperatures.

It is not possible to determine the values of the kinetic constants *k_c_* and *k_d_* describing the processes of formation and decomposition of cluster ions using obtained research results. However, it is possible to estimate the value of the activation energy of the cluster decomposition process. The relationship of the equilibrium constant *K_eq_* and the Gibbs free energy change Δ*G^O^* is described by Equation (14):

(14) $$\Delta G^{O}=\Delta H^{O}-T\Delta S^{O}=-RTlnK_{eq}$$

where Δ*H*^O^ is the standard enthalpy of the reaction and Δ*S^O^* is the entropy change. The standard enthalpy for cluster decomposition is related to the activation energy *E_a_*:

(15) $$-\Delta H^{O}=E_{a}+RT$$

The use of Equations (14) and (15) allows for the determination of the dependence of the logarithm of the equilibrium constant on the reciprocal of temperature:

(16) $$\ln K_{eq}=\frac{E_{a}}{RT}+const$$

The graph of this dependence is shown in Figure 7b. The activation energy of the cluster’s decomposition calculated on the basis of this dependence is 33.2 kJ·mol^−1^, and estimated enthalpy value for the cluster decomposition $-\Delta H_{298}^{O}$ is 35.7 kJ mol^−1^.

## 4. Conclusions

The studies of the drift time spectra carried out with DT IMS while introducing the sample into the drift gas allows for the determination of the kinetic constants and thermodynamic parameters for sample ionization processes. If the carrier gas used in the IMS detector is nitrogen, the ionization of many analytes in the negative mode occurs according to the mechanism of dissociative electron attachment. The sensitivity of detection for such substances is defined by the electron attachment rate constants. The determination of their values is possible by analyzing the shape of the drift time spectra obtained when the sample is introduced into the detector with the drift gas. The values of the electron attachment rate for BzCl that we obtained are in the order of 1 × 10^−8^ cm^3^s^−1^. These values should be recognized as high in comparison to the data contained in the available studies. A slight increase in the value of this constant with temperature is observed. It may be related to the increase in the cross-section for electron capture by BzCl molecules with the increasing electron energy.

The ion movement in the DT IMS drift section can be successfully modeled using differential equations describing the ion balance. These equations take into account diffusion and ion–molecule reactions. Theoretical analysis shows that both phenomena have a significant impact on the shape of the drift time spectra and, in particular, on the peak position, which changes as a result of the processes of formation and decomposition of cluster ions. It can be shown that the phenomenon of peak shifting does not have a significant effect on the accuracy of the determination of the electron attachment rate constant. Measurement of the peak position in the spectrum allows for the determination of the proportion of the average lifetime of chloride and cluster ions and, on this basis, the value of the equilibrium constant for the cluster formation/decomposition processes.

## Figures and Tables

**Table 1:** Drift times and reduced mobilities of ions observed in the drift time spectra measured in the system shown in Figure 1a.

Temperature	Cl^−^ (Chlorine Ions)		M·Cl^−^ (Cluster Ions)	
	*t_d_*, ms	*K*_0_, cm^2^V^−1^s^−1^	*t_d_*, ms	*K*_0_, cm^2^V^−1^s^−1^
318 K	8.25	2.52	12.58	1.65
333 K	7.64	2.60	11.98	1.65
348 K	7.08	2.69	11.44	1.66
363 K	6.64	2.75	No peak	No peak

**Table 2:** Measured values of the electron attachment rates.

Temperature	*k_EC_*, 10^−9^ cm^3^s^−1^		
	C_BzCl_ = 0.92 ppm	C_BzCl_ = 1.84 ppm	C_BzCl_ = 2.75 ppm
318 K	8.09	8.71	9.08
333 K	8.72	9.48	10.20
348 K	9.77	10.50	10.40
363 K	10.80	11.50	11.50

**Table 3:** Equilibrium constants for Equation (2) calculated from the peak shift in the drift time spectra for different BzCl concentrations in the drift gas.

Temperature	*K_eq_*_(2)_		
	C_BzCl_ = 0.83 ppm	C_BzCl_ = 1.88 ppm	C_BzCl_ = 2.94 ppm
318 K	13,630	13,930	14,300
333 K	8780	8790	8880
348 K	4880	4930	5020
363 K	3000	3030	2950

## Data Availability

The data presented in this study are available on request from the corresponding author.

## Supplementary Materials

The following are available online; Modelling of charge transport with MATLAB software.

## Sample Availability

Samples are not available from authors.
