# Supplementary material for: Studies on the Processes of Electron Capture and Clustering of Benzyl Chloride by Ion Mobility Spectrometry
Source: Molecules. 2021 Jul 28;26(15):4562. doi: 10.3390/molecules26154562 (PMC8348954; doi:10.3390/molecules26154562)
Supplement: Supplementary file 1 [file molecules-26-04562-s001.zip › molecules-1293430-supplementary.pdf]

Studies on the Processes of Electron Capture and Clustering of Benzyl Chloride by Ion Mobility Spectrometry

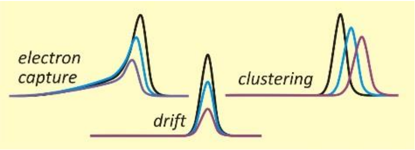

by Izabela Wolańska, Edyta Budzyńska and Jarosław Puton

Supporting information: Modelling of charge transport with MATLAB software

We consider a situation where chloride ions moving in the DT IMS drift section form weakly bound clusters with the molecules of the chemical compound contained in the drift gas. The reaction of the formation and decomposition of clusters is described by the equation:

$$\mathrm{M} + \mathrm{Cl}^- \xleftrightarrow{k_c/k_d} \mathrm{M}\cdot\mathrm{Cl}^- \qquad (1)$$

where k_c is the reaction rate constant for generation of $\mathrm{M{\cdot}Cl^-}$ clusters, and k_d is the constant of their dissociation.

The mathematical description of the movement of ions in the drift section is based on the solution of the so-called the advection-diffusion problem. In the one-dimensional case, the balance of ions moving in the gas under the influence of an electric field corresponds to the system of two partial differential equations:

$$\begin{aligned}\frac{\partial n_{Cl-}}{\partial t} &= -k_c n_M n_{Cl-} + k_d n_{MCl-} - K_{Cl-} E \frac{\partial n_{Cl-}}{\partial x} + D_{Cl-} \frac{\partial^2 n_{Cl-}}{\partial x^2} \\ \frac{\partial n_{MCl-}}{\partial t} &= k_c n_M n_{Cl-} - k_d n_{MCl-} + K_{MCl-} E \frac{\partial n_{MCl-}}{\partial x} + D_{MCl-} \frac{\partial^2 n_{MCl-}}{\partial x^2}\end{aligned} \qquad (2)$$

Where n_{Cl-}, n_{MCl-} and n_M are concentrations of chloride ions, cluster ions and sample molecules, K_{Cl-} and K_{MCl-} mobilities, D_{Cl-} and D_{MCl-} are the diffusion coefficients and E is electric field intensity. The values of the diffusion coefficients can be estimated on the basis of the Nernst-Einstein relationship, taking into account the energy factor η:

$$D = \eta K \frac{k_B T}{e} \qquad (3)$$

Initial conditions necessary to solve the system of equations (2) are the chloride and cluster ion concentration distributions for the time t = 0. This time corresponds to the end of the gating pulse opening the shutter grid. The initial concentration distribution of chloride ions includes two components. The first results from the injection of these ions from the reaction section, and the second corresponds to the ions produced by the capture of electrons introduced into the drift section during the duration of the gating pulse. The initial concentration distribution of the cluster ions includes only the component related to the injection of these ions. The formulas that define the initial conditions are as follows:

$$n_{Cl-}(x,0) = 0.5 n_{0Cl-}\left(erf\left(Bx\right) - erf\left(B(x - K_{Cl-}Et_g)\right)\right) + k_{EC} n_{0e} n_M t_g \exp\left(-\frac{k_{EC} n_M x}{v_e}\right)$$

$$n_{MCl-}(x,0) = 0.5 n_{0MCl-}\left(erf\left(Bx\right) - erf\left(B(x - K_{MCl-}Et_g)\right)\right)$$

(4)

where n_{0e}, n_{0Cl-} and n_{0MCl-} are the concentrations of electrons and respective types of ions introduced through the opened shutter grid, k_{EC} is the electron attachment rate constant, t_g is the shutter grid opening time, v_e is the electron drift velocity and B is the constant determining ion swarm broadening.

Using MATLAB software to solve the equations presented above is simple. Successively follow these steps:
1. call the `CPGenDefault21.m` function that defines parameters for numerical methods (number of points, integration steps, etc.),
2. call the `PPGenDefault21.m` function that defines the physical parameters (temperature, mobility, kinetic constants, etc.),
3. call the main function `AdDi2021.m` that allows to solve the system of equations (2).

A typical structure for commands performed in the MATLAB Command Window is as follows:

```
ADPhParam=PPGenDefault21;
ADCalcParam=CPGenDefault21;
[ion1,ion2,icurr]=AdDi2021(ADPhParam,ADCalcParam);
```

Output variables from `AdDi2021.m` function are `ion1`, `ion2` and `icurr`. `ion1` and `ion2` are the concentration distributions of chloride and cluster ions in consecutive moments of time, and `icurr` is the value of the ion current in the collecting electrode. An example of chloride ions distribution calculated with `AdDi2021.m` function is shown in the figure:

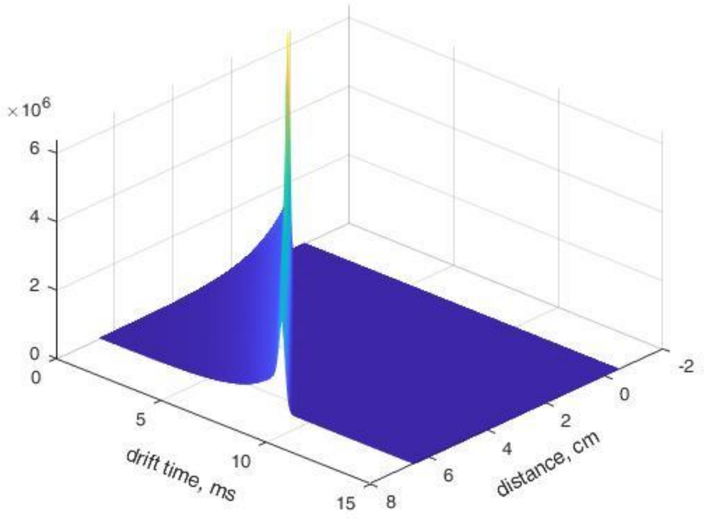

Below are three MATLAB functions that allow you to perform the corresponding calculations. We hope that this description is sufficient for you to be able to make your own calculations. Any clarification can be obtained from JP (jaroslaw.puton@wat.edu.pl).

CPGenDefault21

```
function CalcParam = CPGenDefault
CalcParam.nx=1400;
CalcParam.nt=300;
CalcParam.xmin=-0.5;%cm
CalcParam.xmax=6.5;%cm
```

```
CalcParam.tmax=15.0;%ms
```

PPGenDefault21

```
function PhysParam = PPGenDefault21
PhysParam.K01=2.60;%cm2/Vs
PhysParam.K02=1.65;%cm2/Vs
PhysParam.kEC=7.7e-9;%electron capture constant, cm3/s
PhysParam.kc=1.0e-10;%clusterization constant, cm3/s
PhysParam.kd=2.82e4;%dissociation constant, 1/s
PhysParam.ld=6.10;%drift length, cm
PhysParam.lr=0.4;%length drift gas penetration in reaction section, cm
PhysParam.Acol=1.0;%collector electrode area, cm2
PhysParam.temp=333.0;%K
PhysParam.E=251.0;%V/cm
PhysParam.tg=0.150;%ms
PhysParam.EnFc=3.0;%eta, energetic factor - increasing of D
PhysParam.Berf=20.0;%broadening of the initial peak
PhysParam.Ie0=2.5e-9;%electron current measured @ nMppb=0, A
PhysParam.nMppb=1000.0;%concentration of sample in drift space, ppb
```

AdDi2021

```
function [conc1,conc2,current] = AdDi2021(PhParam,CalcParam)
%SOLUTION of ADVECTION-DIFFUSION PROBLEM with REACTIONS (DESINTEGRATION +
%CLUSTERIZATION). Initial condition contains Cl- ions generated in the
%drift section by electron capture.
%PhParam - structure containing physical parameters. This structure can be:
%generated by function (eg. PPGenDefault21). Particular values of stucture
%components can be edited and changed in workspace.
%CalcParam - structure containing parameters for numerical calculations.
%This structure can be:generated by function (eg. CPGenDefault21).
%Particular values of stucture components can be edited and changed
%in workspace.

% TYPICAL CALLING
%ADPhParam=PPGenDefault21;
%ADCalcParam=CPGenDefault21;
%[ion1,ion2,icurr]=AdDi2021(ADPhParam,ADCalcParam);

global K1 K2 D1 D2 kc kd kEC n0e n01 n02 BroadCoeff temp tgate v1 v2 vele
nM;
temp=PhParam.temp;
K1=PhParam.K01*temp/273.2;
K2=PhParam.K02*temp/273.2;
zpr=temp*PhParam.EnFc/1.1605e4;
D1=K1*zpr;
D2=K2*zpr;
kc=PhParam.kc;
kd=PhParam.kd;
kEC=PhParam.kEC;
v1=K1*PhParam.E;
v2=K2*PhParam.E;
vele=100*(6.78*PhParam.E+2752)*temp/273.2;%in cm/s
ld=PhParam.ld;
lr=PhParam.lr;
Acol=PhParam.Acol;
nesat=PhParam.Ie0/(1.6e-19*vele*Acol);
nM=PhParam.nMppb*2.7e10*273.2/temp;
```

```
n0e=nesat*exp(-kEC*nM*lr/vele);
n01=(nesat-n0e)*vele/(v1+nM*kc*v2/kd);
n02=((nesat-n0e)*vele-n01*v1)/v2;
BroadCoeff=PhParam.Berf;
tgate=PhParam.tg;


nx=CalcParam.nx;
nt=CalcParam.nt;
xmin=CalcParam.xmin;
xmax=CalcParam.xmax;
tmax=CalcParam.tmax;
x=linspace(xmin,xmax,nx);
t=linspace(0.0,0.001*tmax,nt);
m=0;
sol=pdepe(m,@SMpde,@SMic,@SMbc,x,t);
conc1=sol(:,:,1);
conc2=sol(:,:,2);
nld=round(nx*(ld-xmin)/(xmax-xmin));
current(:,1)=1000*t;%in ms;
current(:,2)=1.6e-7*Acol*(conc1(:,nld)*v1+conc2(:,nld)*v2);%in pA;
for i=1:nt
    if current(i,2)<1e-6
        current(i,2)=0;
    end
end
%---------------------------------------------
figure;
plot(current(:,1),current(:,2));
%---------------------------------------------
function [c,f,s] = SMpde(x,t,n,DnDx)
global v1 v2 D1 D2 kc kd nM;
c=[1;1];
f=[D1;D2].*DnDx;
S1=-v1;
S2=-v2;
s=[S1;S2].*DnDx+[-kc*nM;kc*nM].*n(1)+[kd;-kd].*n(2);
%---------------------------------------------
function InCond = SMic(zpx)
global n0e n01 n02 nM BroadCoeff tgate vele v1 v2 kEC;
InCond1=0.5*n01*(erf(BroadCoeff*zpx)-erf(BroadCoeff*(zpx-0.001*tgate*v1)));
xpeak0=0.0005*tgate*v1;
if zpx>xpeak0
    InCond1=InCond1+0.001*n0e*kEC*nM*tgate*exp(-kEC*nM*(zpx-xpeak0)/vele);
end
InCond2=0.5*n02*(erf(BroadCoeff*zpx)-erf(BroadCoeff*(zpx-0.001*tgate*v2)));
InCond=[InCond1;InCond2];
%---------------------------------------------
function [pl,ql,pr,qr] = SMbc(xl,nl,xr,nr,t)
pl=[nl(1);nl(2)];
ql=[0.0;0.0];
pr=[0.0;0.0];
qr=[1.0;1.0];
```
